# Analyzing three pedigrees in X-linked Alport syndrome with the presentation of nephrotic syndrome

**DOI:** 10.3389/fgene.2024.1419154

**Published:** 2024-08-09

**Authors:** Jian-Hui Zhang, Jie Liu, Dan-Dan Ruan, Qian Chen, Jie Yang, Min Wu, Hong-Ping Yu, Li-Sheng Liao, Xiao-Ling Zheng, Jie-Wei Luo, Li Zhang

**Affiliations:** ^1^ Department of Nephrology and Traditional Chinese Medicine, Shengli Clinical Medical College of Fujian Medical University, Fujian Provincial Hospital, Fuzhou, China; ^2^ Department of Digestive Endoscopy, Fujian Provincial Hospital, Fuzhou, China; ^3^ Department of Nephrology, Fujian Provincial Hospital, Fuzhou, China; ^4^ Department of Hematology, Fujian Provincial Hospital, Fuzhou, China

**Keywords:** Alport syndrome, COL4A5 gene, nephrotic syndrome, end-stage renal disease, pedigree analysis

## Abstract

**Background:**

Alport syndrome (AS) is a common cause of end-stage renal disease (ESRD) with various clinical symptoms and incomplete manifestation. Patients with AS and other renal disorders are often misdiagnosed. This study reported three X-linked dominant Alport syndrome (XLAS) pedigrees with nephrotic syndrome (NS) as the predominant phenotype and analyzed *COL4A5* gene alterations.

**Methods:**

Three Han Chinese XLAS pedigrees were recruited, and clinical phenotypes were obtained. The pre-certified individuals’ peripheral blood DNA was taken, and whole-genome next-generation sequencing (NGS) was performed for candidate genes and mutation screening, followed by NGS or Sanger sequencing of suspected mutant types in participating family members.

**Results:**

Both probands A and B were diagnosed with NS through biochemical tests, and X-linked Alport syndrome-associated renal injury was diagnosed by renal biopsy. The biopsy revealed focal foamy cells in the renal interstitium, tearing and delamination changes in the glomerular basement membrane, and negative α3 and α5 chains of type IV collagen. Proband C, who was earlier diagnosed with NS, has now advanced to ESRD, along with his mother and proband A’s mother. Genetic sequencing of all three pedigrees identified three mutations, namely, c.5020C>T, c.4435_4445del, and c.1584_1587+6del in the X-linked dominant gene *COL4A5* (NM_000495.5). These mutations lead to the production of shortened proteins, potentially impacting the function of COL4A5 and causing pathogenic effects.

**Conclusion:**

The novel c.4435_4445del and c.1584_1587+6del mutations not only enrich the spectrum of mutations in the *COL4A5* gene but also indicate that carriers of both mutation sites and those with mutation c.5020C>T may present NS as their primary clinical manifestation.

## Introduction

Alport syndrome (AS) is a prevalent genetic kidney disease known as hereditary nephritis and oculo-auriculo-renal syndrome. It is caused by mutations in the *COL4A3*, *COL4A4*, and *COL4A5* genes, which encode the α3, α4, and α5 chains of type IV collagen. X-linked dominant, autosomal recessive, or autosomal dominant inheritance are the modes of inheritance of AS, which causes hematuria, proteinuria, sensorineural deafness, ocular abnormalities, and kidney damage that leads to end-stage renal disease (ESRD) (Daga et al., 2022). X-linked Alport syndrome (XLAS, OMIM: 301050) is caused by a pathogenic variant in the *COL4A5* gene and is the most common mode of inheritance. Men have up to a 100% risk of progression to ESRD, and the age of progression and extrarenal manifestations correlate with the genotype; women have a 25% risk and an increased risk with age (Alport Syndrome Collaborative, National Clinical Research Center of Kidney, & Rare Diseases Branch of Beijing Medical, 2023). A history of childhood hematuria, steadily deteriorating proteinuria, and sensorineural deafness are risk factors for the advancement of this illness (Jais et al., 2003).

Patients with AS can exhibit various clinical symptoms, which may not include the typical signs during the initial stages. Consequently, when AS occurs alongside other kidney diseases, patients may be mistakenly diagnosed with IgA nephropathy, nephrotic syndrome (NS), purpura nephritis, or thin basement membrane nephropathy (Deng, Zhou and Zhou, 2023). Cases of AS with NS as its primary symptom are infrequently documented, leading to the potential oversight of AS and subsequent delays in diagnosis, ultimately culminating in the progression to ESRD. This article presents three pedigrees of XLAS resulting from variations in the *COL4A5* gene, with the identification of two novel mutation sites. Unexpectedly, all three probands presented with NS as their primary clinical symptom, with two of them experiencing NS as their initial symptom. We conducted genetic and phenotypic analyses of the three pedigrees, highlighting the crucial nature of renal pathological examination and genetic testing in diagnosing atypical kidney diseases.

## Method and material

### Research subjects

**FIGURE 1:**
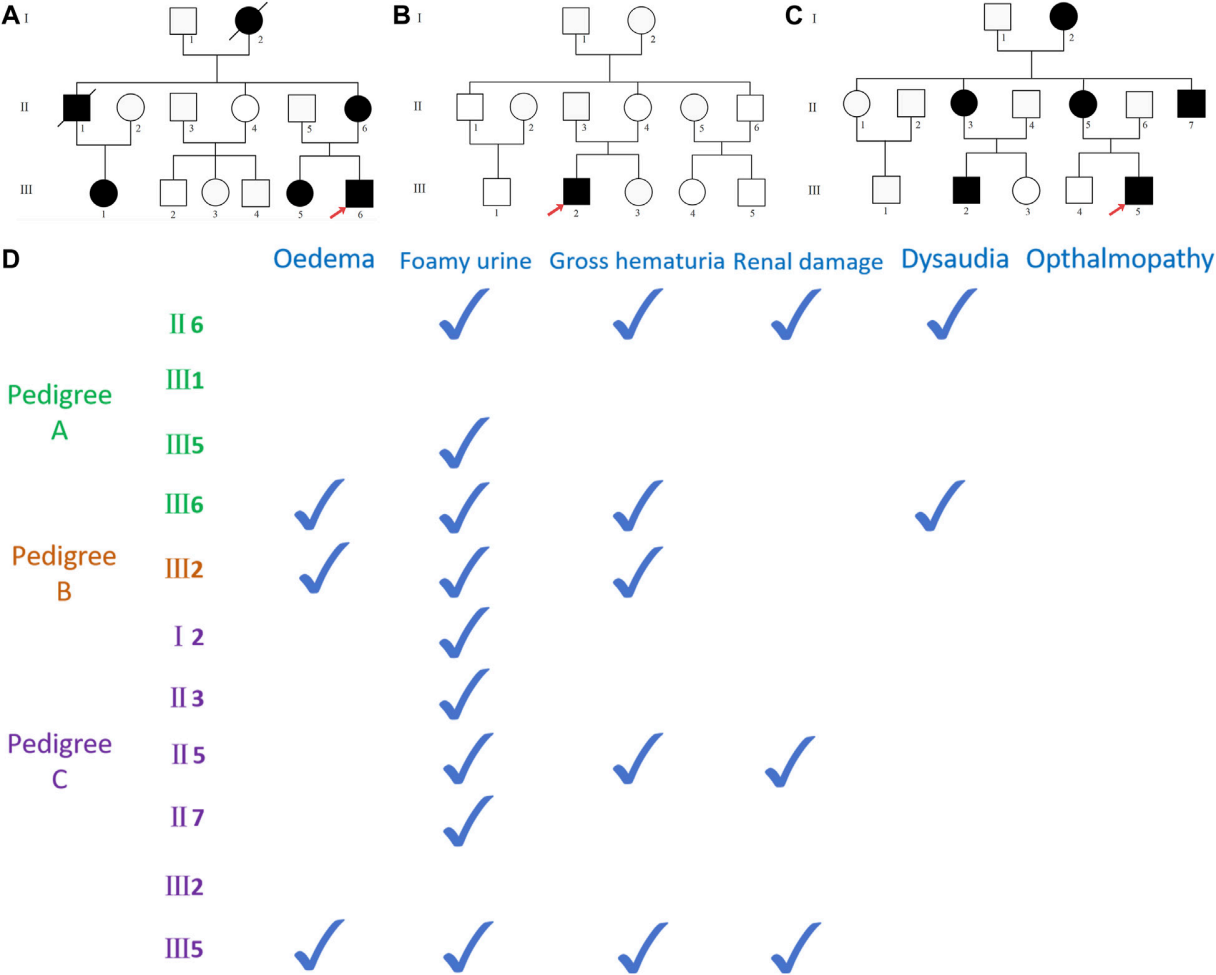
**(A)** Pedigree A map, black representing carriers of the *COL4A5* (NM_000495.5) c.5020C>T mutation, with red arrows representing pre-documented individuals, squares representing male population, and circles representing female population; **(B)** pedigree B map, black representing carriers of the *COL4A5* (NM_000495.5) c.4435_4445del mutation; **(C)** pedigree C map, black representing *COL4A5* (NM_000495.5) c.1584_1587+6del mutation carriers. **(D)** The documented presence of clinical symptoms in the carriers of the mutant in three pedigrees.

Three Chinese pedigrees with Alport syndrome were collected in the study: 12 people from pedigree A, 13 people from pedigree B, and 14 people from pedigree C. Within the pedigrees, there were no consanguineous marriages (Figures 1A–C). The Fujian Provincial Hospital Ethics Committee approved the study. The pedigree members willingly signed an informed consent form before the clinical investigation. Proband A (A-III6) and proband B (B-III2) experienced a sudden onset of illness, characterized mostly by edema of the eyelids and both lower limbs, together with hematuria and foamy urine, but with normal urine output. When proband C (C-III5) was 3 years old, he began to exhibit unclear-source hematuria, and the renal pathology at that time pointed to diffuse proliferative glomerulonephritis with a few segmental scleroses. At the age of 17 years, he suddenly developed swelling of the eyelids and the lower limbs, with serum albumin of 20 g/L and a large amount of proteinuria, which was diagnosed as “nephrotic syndrome.” A second renal biopsy suggested proliferative glomerulonephritis with focal and segmental glomerulosclerosis (FSGS), and he has been on maintenance peritoneal dialysis for more than 10 years because of his ESRD. Probands A and C, whose mothers (A-II6 and C-II5) are presently receiving hemodialysis for ESRD, have a medical history of frothy urine and hematuria. Apart from a few members of pedigree A (II6 and III6), none of the respondents had hearing impairment, and none of the pedigrees had reported ocular pathology. Other members of the three pedigrees were examined for past and present disease histories, as well as for general physical examinations, alongside genetic testing (Figure 1D).

### Methods

#### DNA extraction

Peripheral blood samples (5 mL) were taken from the proband and family members and collected in an anticoagulant tube using ethylenediaminetetraacetic acid (Shanghai Solarbio Bioscience & Technology Co., Ltd., Shanghai, China), and the genome was extracted using the QIAamp DNA Blood Mini Kit (QIAGEN, Germany). DNA concentration and purity were measured using a NanoDrop 1000 Spectrophotometer (NanoDrop Technologies, United States).

#### Candidate gene location and mutation-screening strategies

After testing for DNA quality, whole-genome sequencing was performed, and based on bam results of comparison with genome reference sequences, SAMtools, GATK, and ANNOVAR were used to find and analyze SNVs and indels in the sequencing data and screen and annotate variants according to ACMG (Richards et al., 2015) in databases such as dbSNP, 1000G, HGMD, and ESP6500. Sequence reads were compared with hg19 sequences using BWA to analyze gene information, mutation types, frequencies from databases such as 1000G and ESP6500. Mutation pathogenicity was predicted using software like PolyPhen-2, Sorting Intolerant from Tolerant, and MutationTaster. Primer Premier 5.0 was used to create amplification primers for the upstream and downstream regions of sequences with target mutation sites, enabling the amplification of target regions.

#### Sanger sequencing verification

PCR amplification and Sanger sequencing were used to validate the candidate mutation sites in the proband and the pedigrees. Using the GenBank (NM_000495.5) *COL4A5* gene sequence, Primer Premier5.0 software designed primers for the target sequences. The primers were synthesized by Synbio Technologies Co., Ltd. (Suzhou) (Table 1). PCR products were amplified and purified using the reagent from TaKaRa (Takara Biomedical Technology Co., Ltd., Beijing) and sequenced using an ABI 3730XL sequencer (Beijing Jingkreida Technology Co., Ltd., Beijing) to detect the PCR products of target fragments.

**TABLE 1:** Primer design of the fragments with suspected responsibility point mutation of each Alport syndrome pedigree.

Pedigree	Mutation	Exon	Forward primer	Forward primer	Length (bp)
B	c.4435_4445del	Exon47	TTT​TAT​TTG​TCT​CCT​AGC​CCA​T	TTA​GGC​CAA​GAA​TTG​TAC​CTC​A	610
C	c.1584_1587+6del	Exon23	ATG​GGA​TTG​AAT​GGG​GTT​CTT	CTC​ACA​GGC​TCT​CCT​TTC​GG	713

## Results

### Clinical phenotypes

**FIGURE 2:**
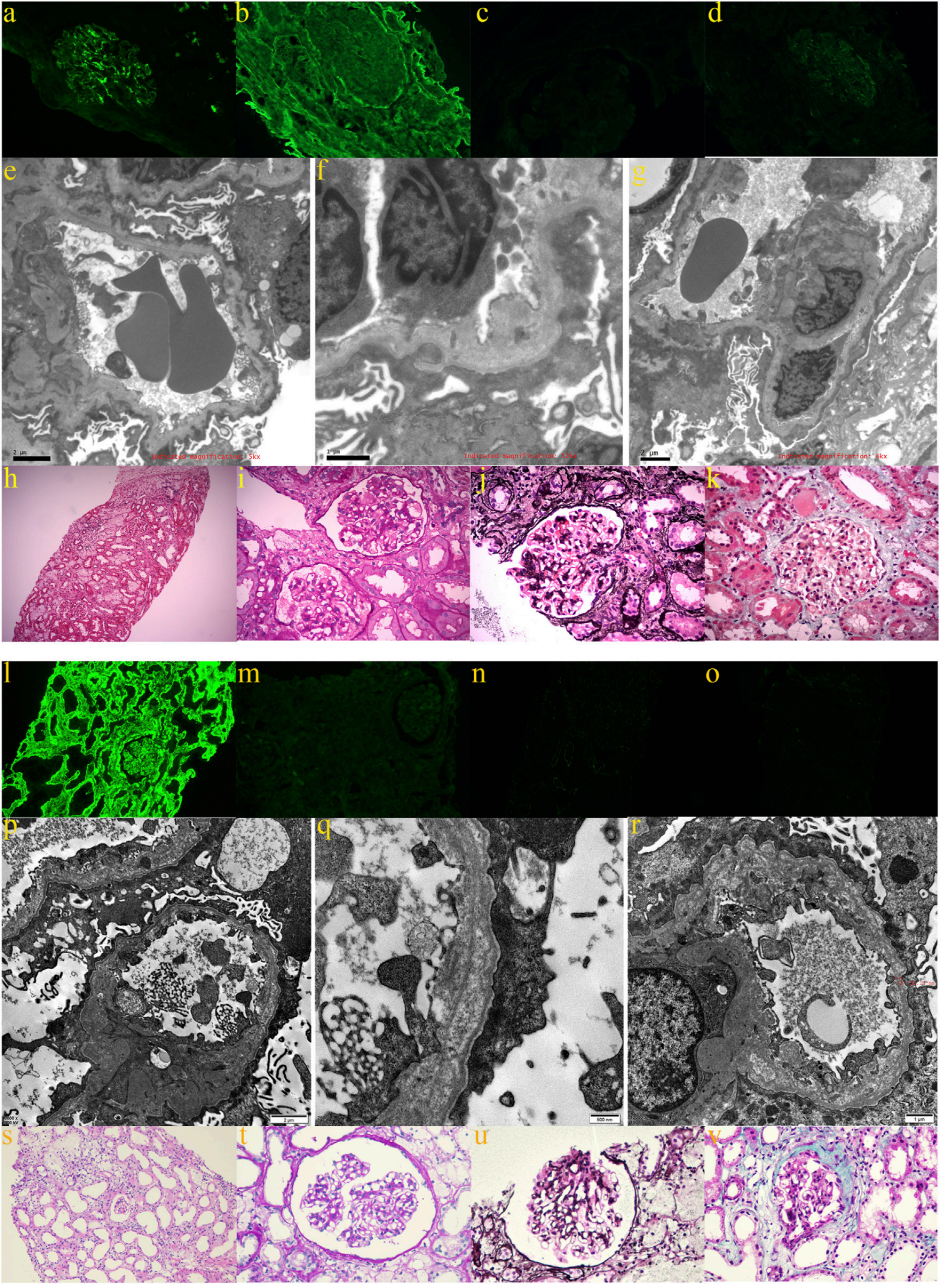
Renal pathological findings of proband A: **(A)** weakly positive immunofluorescence with IgA; **(B)** positive type IV collagen α1 staining; **(C)** negative type IV collagen α3 staining; **(D)** negative type IV collagen α5 staining; **(E–G)** electron microscopy revealed glomerular basement membranes of varying thicknesses and thinnesses, basement membrane tearing, layered alterations, a diffuse fusion of podocyte foot processes, and mild proliferation of the glomerular mesangial cells and stroma; **(H,I)** light microscopy using H&E and PAS staining revealed mild hyperplasia of some glomerular mesangial cells and stroma, vacuolar degeneration of renal tubular epithelial cells, small foci of inflammatory cell infiltration in the renal interstitial, and foci of foamy cells (200×; 400×); **(J)** PASM staining did not reveal peg-like structures, thylakoid insertion, or double track formation (400×); **(K)** Masson staining did not reveal a distinct lesion (400×). The renal pathological findings of proband B: **(L)** positive immunofluorescence α1; **(M)** negative type IV collagen staining α3; **(N)** negative type IV collagen staining α4; and **(O)** negative type IV collagen staining α5; **(P–R)** electron imaging revealed the stratification of the dense layer of the glomerular basement membrane with incomplete inner and outer rims in the form of a flower basket and proliferation of the glomerular tethered area of the tethered cells and stroma; **(S,T)** the glomerular capsule wall thickened, and extracapsular fibrosis, vacuolar and granular degeneration of renal tubular epithelial cells, multiple tubular foamy degeneration, visible protein tubular pattern, interstitial edema, focal inflammatory cell infiltration, diffuse clusters of foamy cell infiltration, and mild fibrosis of the interstitium were all revealed by light microscopic H&E and PAS staining (200× and 400×); **(U)** PASM staining failed to reveal peg-like structures without tethered membrane insertion and double-track formation (400×); **(V)** Masson’s staining did not reveal any obvious lesions (400×).

Proband A (A-III6) and proband B (B-III2) both exhibited hypoproteinemia and massive proteinuria (Table 2), and they were diagnosed with nephrotic syndrome. Proband C (C-III5) and his mother (C-II5) and proband A’s mother (A-II6) had glomerular filtration rates below 10 mL/min, leading to the diagnosis of ESRD. Additionally, they experienced renal anemia and serum electrolyte disorders (Table 2). Furthermore, proband A’s grandmother (A-Ⅰ2) and uncle (A-II1) succumbed to renal illness. Renal-related biochemical markers did not exhibit any abnormalities in the remaining members of pedigrees A and C (A-III1, A-III5, C-II3, C-II7, and C-III2). Probands A and B underwent ultrasound-guided percutaneous kidney puncture biopsies with informed consent. Immunofluorescence analysis of renal pathology in proband A showed IgM positivity, weak positivity for IgA and C3, negativity for IgG and C1q, and positivity for type IV collagen staining α1, but negativity for α3 and α5(Figures 2A–D). Electron microscopy revealed that the glomerular basement membrane exhibited varying thickness, ranging from approximately 150 to 390 nm. Additionally, the membrane displayed tears, layered alterations, a diffuse fusion of podocyte foot processes, mild hyperplasia of the tethered cells, and stroma. Notably, there was an absence of electron-dense material in the subepithelial region, interior of the basement membrane, or subendothelial area. The renal tubular epithelial cells display vacuolar degeneration, together with a minor presence of inflammatory cells in the renal interstitium (Figures 2E–G). A light microscopic stain showed a small amount of photophilic protein deposition in the mesangial area, inadequate capillary collateral dilatation, uneven basement membrane staining, and modest hyperplasia of certain glomerular mesangial cells and stroma. Renal tubular epithelial cells, vacuolar degeneration, focal inflammatory cell infiltration restricted by the renal interstitium, no discernible fibrosis, focal foam-like cells visible in the renal interstitium, and no visible lesions in the small arteriolar wall (Figures 2H–K). Immunofluorescence analysis of proband B suggested weak positivity for IgM and C3, negativity for IgG, IgA, C1q, Fib, and ALB, and positivity for α1; α3, α4, and α5 expressions were absent (Figures 2L–O). Electron microscopy discovered that the thickness of the glomerular basement membrane varied between 100 and 300 nm. The dense layer of the membrane had a stratified structure, with incomplete inner and outer edges resembling a flower basket. There was an increase in the number of mesangial cells and stroma in the area where the glomerulus is attached. The podocyte foot process pedicles showed widespread fusion. Some glomerular attachment sites contained a small amount of electron-dense material. Additionally, there was metaplasia of the vacuoles in the tubular epithelial cells. No distinct abnormalities were observed in the renal interstitium (Figures 2P–R). Extracapsular fibrosis and thickening of the glomerular capsule wall were visible under light microscopy. The features that are visible in the renal tubules include dilated lumens in most of them, brush border loss, diffuse clusters of foamy cell infiltration, renal tubular epithelial cell vacuolation, granular degeneration, multiple tubular foamy degenerations, visible protein tubular patterns, renal interstitial edema, and mild fibrosis of the interstitium (Figures 2S–V). The renal biopsies of probands A and B revealed the presence of foam-like cells in the renal interstitium and ripping and layering abnormalities in the glomerular basement membrane. Additionally, the staining for type IV collagen was negative for both α3 and α5; therefore, XLAS renal injury was considered.

**TABLE 2:** Clinical data of the members in every family associated with Alport syndrome.

Item	Pedigree A		Pedigree B	Pedigree C		Normal value
	Ⅱ6	Ⅲ6 (proband A)	Ⅲ2 (proband B)	Ⅱ5	Ⅲ5 (proband C)	
Basic information						
Sex	Female	Male	Male	Female	Male	-
Age (years)	50	17	16	53	31	-
First discovery (age and years)	45	17	16	51	3	-
ESRD	Y	N	N	Y	Y	N
Blood routine tests						
WBC (×10^9^/L)	7.8	9.0	5.6	6.8	7.3	4.1∼11.0
RBC (×10^12^/L)	2.26	4.35	4.23	2.93	3.25	4.10∼5.80
Hb (g/L)	64	126	133	85	97	114∼154
PLT (×10^9^/L)	160	273	218	146	208	150∼407
Urine tests						
Protein	N/A	(+++)	(+++)	(+)	N/A	(−)
24-h proteinuria (g/24 h)	N/A	7.904	10.589	N/A	N/A	0.05∼0.1
RBC (/μL)	N/A	452.4	171	57.8	N/A	0∼13.1
Biochemical indexes						
TP (g/L)	63	43	39	67	55	65∼85
ALB (g/L)	31	23	23	36	25	40∼55
GLB (g/L)	32	20	16	31	30	20∼40
TC (mmol/L)	4.86	6.02	9.55	2.77	5.43	<5.2
TG (mmol/L)	0.66	1.58	1.74	1.43	1.16	<1.7
CK (U/L)	487	170	152	26	159	40∼200
BUN (mmol/L)	19.4	7.0	6.1	34.4	17.2	3.1∼8.8
Scr (μmol/L)	1,082	58	72	822	1,018	41∼81
GFR (mL/min)*	6.0	294.5	232.3	8.1	9.6	80∼120
UA (μmol/L)	415	338	340	285	450	155∼357
Glucose (mmol/L)	4.37	5.61	5.39	4.32	7.70	3.9∼6.1
Na (mmol/L)	138	141	139	134	139	137∼147
K (mmol/L)	4.5	4.0	4.0	3.9	3.7	3.5∼5.3
Cl (mmol/L)	98	109	107	98	97	99∼110
Ca (mmol/L)	2.45	1.97	2.12	2.29	2.25	2.11∼2.52
Mg (mmol/L)	0.69	0.73	0.82	1.04	0.68	0.75∼1.02
P (mmol/L)	1.64	1.44	0.96	2.51	1.79	0.85∼1.51

Note: ERSD, end-stage renal disease; WBC, white blood cell count; RBC, red blood cell count; Hb, hemoglobin; PLT, platelet count; TP, total protein; ALB, albumin; GLB, globulin; TC, total cholesterol; TG, triglyceride; BUN, serum urea nitrogen; Scr, serum creatinine; UA, uric acid; Na, serum sodium; K, serum potassium; Cl, serum chloride; Ca, serum calcium; Mg, serum magnesium; P, serum phosphate. Y, yes; N, none; (+), positive; (−), negative. *GFR by MDRD clearance (ml/min).

### Screening for mutations in the Alport syndrome gene

**FIGURE 3:**
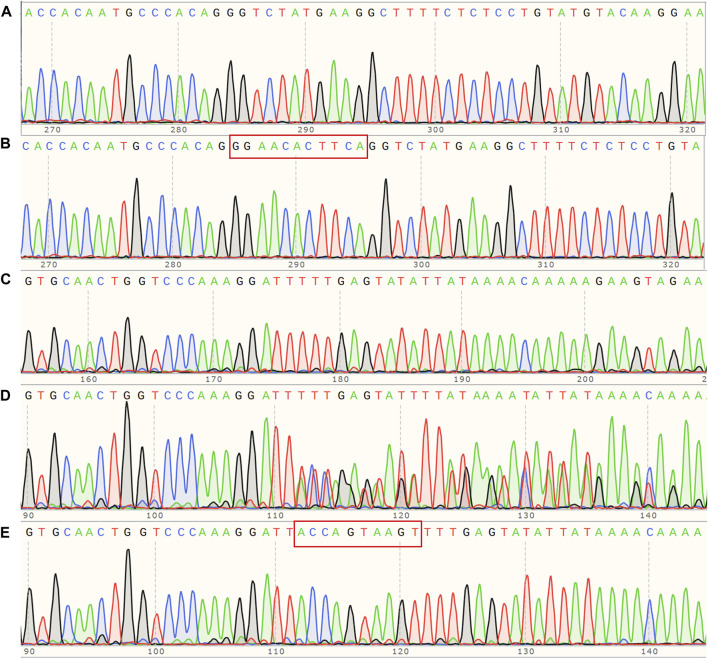
**(A)** Sanger sequencing map of pedigree B, *COL4A5* (NM_000495.5) c. 4435_4445del hemizygous mutant; **(B)** corresponding wildtype of pedigree B; **(C)** Sanger sequencing map of pedigree C, *COL4A5* (NM_000495.5) c.1584_1587+6del hemizygous mutant; **(D)** corresponding heterozygous mutant; **(E)** corresponding wild type.

Each proband had one mutant site and was hemizygous with X-linked dominant inheritance according to whole-genome sequencing. Proband A has the mutation site c.5020C>T in exon53 of *COL4A5* (NM_000495.5), which turns the arginine at position 1,674 into a termination codon (p.Arg1674Ter) and truncates the protein. Proband B has a deletion mutation in exon47 of *COL4A5* (NM_000495.5) that produces p.Thr1480Leufs* 2, caused by the deletion of 11 bases from positions 4,435 to 4,445. Proband C has a deletion mutation in exon23 of *COL4A5* (NM_000495.5) that deletes bases 1,584–1,587 and six intronic bases (c.1584_1587+6del), causing the termination codon to occur early and creating a shortened protein. Whole-genome sequencing was performed on all members of pedigree A upon special request. The results showed that A-II6, A-III1, A-III5, and A-III6 carry the mutation c.5020C>T. Given that this mutation (p. Arg1674Ter) has been reported and considered pathogenic (Zhang et al., 2018), we hypothesize that the two members of the pedigree who passed away from renal disease (A-Ⅰ2 and A-Ⅱ1) may also carry this mutation. The remaining pedigree B and C members were Sanger-sequenced. In pedigree B, only the proband has the c.4435_4445del (p.Thr1480Leufs* 2) mutation, while the rest of the members are wild type (Figures 3A, B). In pedigree C, Ⅰ2, Ⅱ3, Ⅱ5, Ⅱ7, Ⅲ2, and Ⅲ5 carry the c.1584_1587+6del mutation, while in others, no mutants were found (Figures 3C–E). As per the ACMG (Richards et al., 2015), the mutation c.5020C>T is classified as pathogenic based on the criteria PVS1_Strong + PS4_Supporting + PM2_Supporting. Similarly, the mutation c.4435_4445del is also considered pathogenic according to the criteria PVS1_Strong + PS2 + PM2_Supporting. On the other hand, the mutation c.1584_1587+6del is classified as possibly pathogenic based on the criteria PVS1 + PM2_Supporting.

### Prediction and analysis of bioinformatics

**FIGURE 4:**
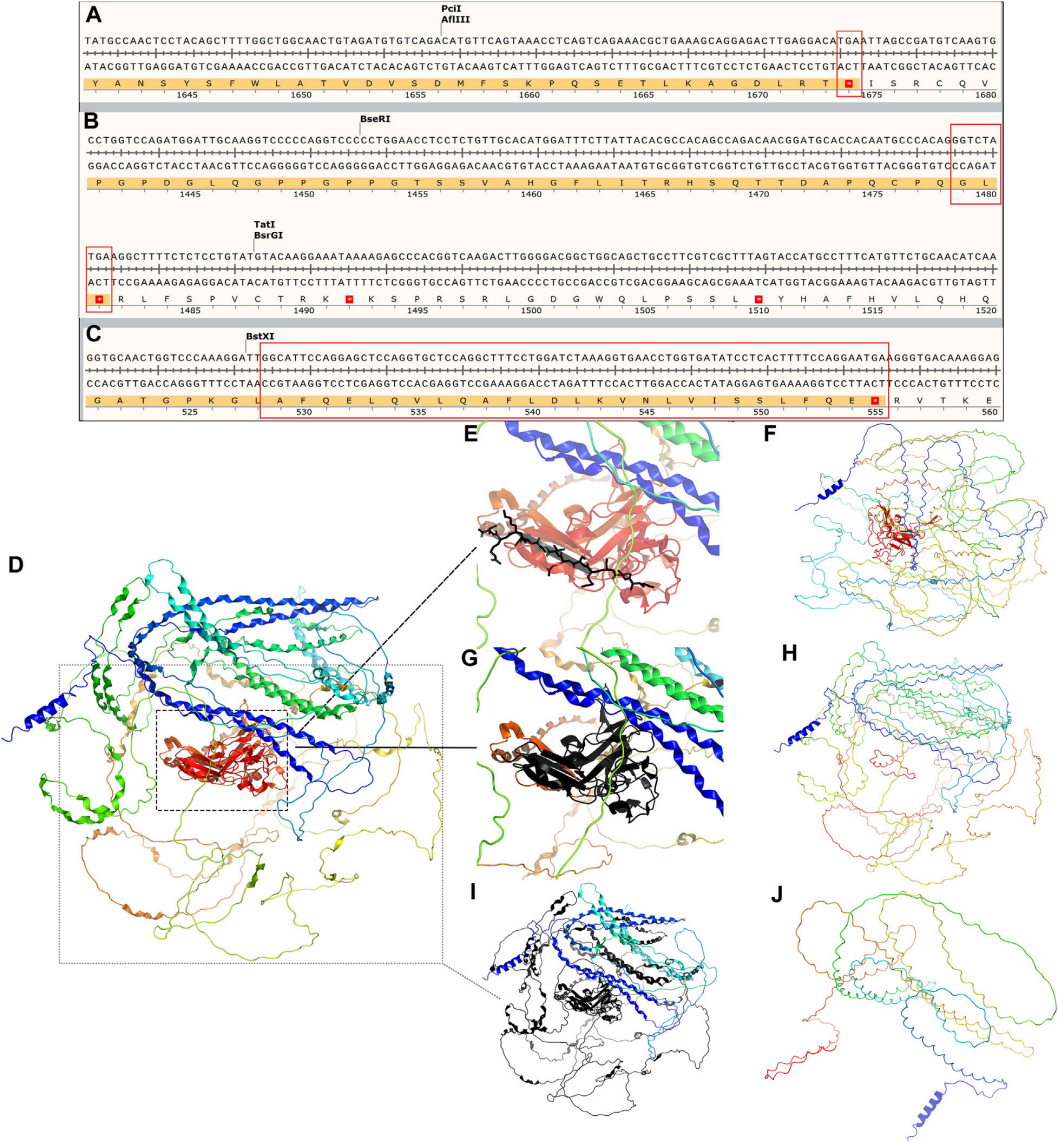
**(A–C)** Amino acid sequence changes following the three mutations c.5020C>T, c.4435_4445del, and c.1584_1587+6del of *COL4A5* (NM_000495.5), all of which lead to an early termination codon and end of translation of the protein COL4A5. The tertiary structure of COL4A5 wildtype and mutants were predicted using SWISS-MODEL (https://swissmodel.expasy.org/repository/uniprot/P29400) and shown in PyMOL. **(D)** Tertiary structure of COL4A5 wildtype protein; **(E)** black part represents the protein structure of mutant c.5020C>T deletion; **(F)** tertiary structure of mutant c.5020C>T protein; **(G)** black represents the protein structure of mutant c.4435_4445del deletion; **(H)** tertiary structure of mutant c.4435_4445del protein; **(I)** black part represents the missing protein structure of mutation c.1584_1587+6del; **(J)** protein 3D structure of mutant c.1584_1587+6del.

Our investigation revealed that all three mutants generate shortened proteins (Figures 4A–C) due to the premature occurrence of the stop codon, and all of them are classified as pathogenic. To verify this hypothesis, we employed SWISS-MODEL (https://swissmodel.expasy.org/repository/uniprot/P29400) to forecast the 3D configurations of the proteins belonging to the wild-type (WT) and COL4A5 mutations. Subsequently, we displayed the three-dimensional structure image of the COL4A5 WT using PyMOL (Figure 4D). Despite losing fewer sequences (black depicts the mutant’s missing section) (Figure 4E), the protein’s three-dimensional structures have changed (Figure 4F). c.4435_4445del and c.1584_1587+6del mutants lack additional sequences (Figures 4G, I). Mutated proteins had very different three-bit structures (Figures 4H, J).

## Discussion

As estimated, 1/10,000–1/5,000 people have AS, which accounts for 0.5% of newly diagnosed adult cases of ESRD (Mallett et al., 2014). Nonetheless, it was shown that COL4A5 variants with potential for disease were often found in renal failure queues and normal reference data, suggesting that the occurrence rate of XLAS was around 1/5,000 (Groopman et al., 2019; Connaughton and Hildebrandt, 2020). This supports AS being the second leading cause of hereditary renal failure after autosomal dominant polycystic kidney disease (Savige et al., 2021). Nephrotic syndrome is a rare but dangerous kidney illness that affects people worldwide, including adults and children. Edema, proteinuria, hypoalbuminemia, and hyperlipidemia are common symptoms (C. S. Wang and Greenbaum, 2019). Incidence rates are 3 per 100,000 adults and 2–7 per 100,000 children annually. Although it occurs rarely, it is responsible for 20% of pediatric ESRD cases and roughly 12% of all ESRD cases (Verma and Patil, 2024). Numerous conditions can lead to NS, including primary glomerulonephritis, infections, tumors, and drug-induced illnesses. In our pedigree study, all three probands met the diagnostic criteria for the nephrotic syndrome: albumin levels less than 30 g/L, 24-h urine protein levels more than 3.5 g/L, edema, and/or high lipid levels (Kodner, 2016). We did not delay performing renal puncture for the two patients with AS whose first manifestation was nephrotic syndrome; however, proband C was not diagnosed with AS in a timely manner, probably because of limited medical technology or a lack of awareness of AS at that time. He was also not found to carry the *COL4A5* mutant until he progressed to ESRD by genetic testing.

Nephrotic syndrome primarily arises from the impairment or malfunction of glomerular components, such as the glomerular basement membrane, endothelial surface, or epithelial cells (podocytes) (Politano, Colbert, and Hamiduzzaman, 2020). This results in the excretion of protein in the urine and encompasses three primary types of pathology: microscopic lesion disease, FSGS, and idiopathic membranous nephropathy. Among these, FSGS is the primary cause of ESRD in nephrotic syndrome (Ozeki et al., 2021). Furthermore, AS leads to the development of either segmental or widespread mesangial cell hyperplasia, accompanied by an increase in mesangial stroma as the disease advances, and some cases may present glomerulopathy with FSGS (Alport Syndrome Collaborative et al., 2023). Proband C’s second renal pathology was diagnosed as mesangial proliferative glomerulonephritis with FSGS, which aligns with AS-related renal pathology. The patient experienced prolonged hematuria starting at the age of 3, which is more likely to be attributed to an inherited nephrotic disease-related nephrotic syndrome in a pediatric patient with a family history of the disease. In the remaining two probands, electron microscopy revealed tearing and layering alterations in the basement membrane of the kidneys. Additionally, immunofluorescence analysis showed a lack of α3 and α5 collagen staining, which aligns with the characteristic renal damage observed in individuals with AS. Therefore, both patients can be classified as having AS-related nephrotic syndrome. What sets these previous patients apart is that they not only exhibit the classic symptoms of nephrotic syndrome, but they also have hematuria. Hematuria can be a significant clinical sign of nephrotic syndrome when combined with AS. A case report from China details the presentation of an 11-month-old infant with AS who primarily exhibited nephrotic syndrome (D. Wang et al., 2021). Additionally, the child experienced extensive hematuria, but her renal function remained unaffected. Another Chinese child diagnosed with AS exhibited nephrotic syndrome as the major symptom, along with the presence of microscopic hematuria (Deng et al., 2023). Therefore, we should not assume that hematuria is solely caused by nephrotic syndrome since it may serve as a crucial indicator in our investigation of the underlying cause of nephrotic syndrome.

Undoubtedly, type IV collagen α-chain component anomalies in probands A and B necessitate genetic testing. Furthermore, patients should have undergone *COL4A5* genetic testing if they have persistently abnormal hematuria for more than 6 months or persistent proteinuria greater than 0.5 g per day in addition to renal biopsy confirmation of FSGS (Savige et al., 2022), so proband C should have undergone genetic testing much earlier. Through genetic testing, it was determined that all three probands were XLAS patients. Among male XLAS patients, there was a significant correlation between their genetic makeup and the severity of their kidney symptoms. Patients with truncating variants, such as nonsense variants or small insertions/deletions that cause premature termination of codons, exhibited more severe symptoms compared to patients with missense variants or small in-frame variants (Yamamura et al., 2020). As of the current date (2024-03-14), the ClinVar (https://www.ncbi.nlm.nih.gov/clinvar) database includes 192 frameshift mutations and 113 nonsense mutations, all of which are classified as pathogenic or likely pathogenic. Out of the three mutations we found, mutation c.5020C>T is harmful and associated with bilateral symmetrical sensorineural deafness (Zhang et al., 2018). Coincidentally, both proband A and his mother, who also has the mutation c.5020C>T, have a simultaneous combination of hearing impairment. Patients with missense mutations have a 60% risk of hearing loss by the age of 30, while patients with other types of mutations have a 90% risk (Jais et al., 2000). Most patients with hearing loss experience mild to moderate hearing loss, which worsens over time. The other two families, although currently unaffected, will need ongoing hearing tests. The mutation sites c.4435_4445del and c.1584_1587+6del have not been previously documented. The ACMG recommendations classify c.4435_4445del as pathogenic and c.1584_1587+6del as probabilistically pathogenic. We conducted a three-dimensional protein structure analysis and discovered that both mutations result in the production of shortened proteins. The region that is absent encompasses not just the specific mutation site c.5020C>T but also extends beyond it. Therefore, we hypothesize that both mutations may have an impact on the protein’s function and are likely to be pathogenic.

Currently, there is no definitive remedy for AS. The treatment primarily aims to decrease proteinuria and slow down the advancement of kidney disease. Although treatment can postpone the start of renal impairment, most AS patients will eventually require dialysis or renal transplantation. For men diagnosed with XLAS, it is advisable to start renin–angiotensin–aldosterone system (RAAS) blocker medication (Kashtan and Gross, 2021). Probands A and B were instructed to take oral valsartan and dapagliflozin to decrease the excretion of protein in the kidneys. Furthermore, both patients had the additional condition of nephrotic syndrome. After taking glucocorticoids, there was a considerable improvement in edema for both patients, and adjuvant medications such as compound α-ketoacid, calcium, gastric preservation medication, and atorvastatin were utilized. Moreover, the two probands’ continued to experience hematuria while showing improvement in proteinuria during the follow-up. The fact that glucocorticoid therapy usually has no effect on nephrotic syndrome associated with Alport syndrome (D. Wang et al., 2021) leads us to consider that the two patients may have combined minimal change nephrotic syndrome. Proband C and the mother of both probands had reached ESRD and needed renal replacement treatment.

## Conclusion

New mutations c.4435_4445del and c.1584_1587+6del enriched the *COL4A5* gene mutation spectrum, and carriers of these two mutation sites and c.5020C>T may present nephrotic syndrome as the predominant clinical symptom. Renal pathological examination and genetic testing are crucial to diagnosing AS when NS is the major indication. This is because the standard renal signs are lost, making it easy to detect or misdiagnose.

## Data Availability

The datasets presented in this study can be found in online repositories. The names of the repository/repositories and accession number(s) can be found at: https://www.ncbi.nlm.nih.gov/, SCV004698203 https://www.ncbi.nlm.nih.gov/, SCV004698204 https://www.ncbi.nlm.nih.gov/, SCV004698332.
